# Supplementary material for: Effect of underwater visual survey methodology on bias and precision of fish counts: a simulation approach
Source: PeerJ. 2018 Jul 30;6:e5378. doi: 10.7717/peerj.5378 (PMC6071614; doi:10.7717/peerj.5378)
Supplement: Article S1 — Detailed description of the model according the the ODD protocol (Overview, Design concepts, Details) for describing agent-based models. [file peerj-06-5378-s001.pdf]

S1. FishCensus ODD Description

Miguel Pessanha Pais (mppais@fc.ul.pt)

MARE – Marine and Environmental Sciences Centre, Faculdade de Ciências, Universidade de Lisboa, Portugal.

The model description follows the ODD (Overview, Design concepts, Details) protocol for describing individual-based models (Grimm et al., 2010, 2006). This description applies to model version 2.0 (available at the CoMSES repository).

1) Purpose

The model simulates how different fish behaviours affect density estimates in common underwater visual census methods. These include accuracy and precision of estimates and bias due to non-instantaneous sampling. Ultimately, the model can help decide the best method and sampling effort for a real upcoming field assessment.

2) Entities, state variables, and scales

2.1) Landscape and scales

The model is spatially explicit and has two types of moving agents, divers and fish. Agents are spatially represented by their correct size for scale purposes, but spatial interactions treat agents as points (distances are measured between agent coordinates, regardless of size). The model landscape is represented by a grid of squares with 1 m sides that have no variables directly affecting agents. Depth is ignored (assumed constant) and maximum underwater visibility was set to 6 meters and remained constant in time and space. The landscape size was set to 20 x 80 squares (1600 m^2). Divers and fish wrap around when they reach the edges to avoid artificial gatherings near walls. The origin of the coordinate system is located in the centre of the bottom left square, so that integer coordinate values always correspond to square centres, even though agents move in continuous space.

There are two levels on the time scale. Fish and diver movements use a time step representing 1/10 of a second and all other procedures in the model are based on a time step of one second. Model runs stop when finishing conditions are met for the sampling method chosen (i.e. diver reaches a fixed distance or sampling time).

2.2) Diver

The diver is a single agent, responsible for performing a chosen sampling method with pre-defined input parameters. Parameters for fixed distance transects are diver speed (in meters per minute), transect width (m) and transect distance (m). For stationary

point count samples, parameters are turning speed (degrees per second), sampling radius (m) and total sampling time (minutes). Diver state variables are their x and y coordinates, heading (degrees) and a constant speed (meters per minute). There is a fixed view angle value that defines a field of view in front of the divers and is set to 180 degrees for transects and 160 degrees for stationary points and random path (Ward-Paige et al., 2010). For the fixed distance transect, the initial and final y coordinates are recorded as diver attributes, and are used to check when to finish sampling.

The information about observed fishes is stored in 3 diver variables. One is a list of unique ID numbers of counted fishes, which represents a visual memory and prevents recounts, the second one is a snapshot list of the “species” attribute of every fish inside the sampling area on the first time step (i.e. a “snapshot” of the sample area) and the third one is the list of the “species” attribute of every counted fish appended as the sampling method is simulated.

Besides maximum visibility, counts are affected by two limiting parameters, count saturation and memory. Count saturation is the maximum number of fish that a diver can register in a second, with priority given to closest fish. This is set to 3 fish based on visual working memory studies (Luck and Vogel, 1997). Fish in the memory list are not recounted, however, fish that leave the field of view (delimited by view angle and maximum visibility) have their IDs removed from the diver’s memory list and can be recounted.

For every method, a second diver (buddy) can be deployed and will simply move with the main diver, staying 1 meter behind and 1 meter to the right. This diver does not count fish and has no other attributes, but triggers evasive behavior on fish.

2.3) Fish

Fish are the most complex entities in the model. Within fish agents, there can be different “species” that share the same attributes and size (total length in meters). Besides species name and size, there are several other attributes of fish agents that stay constant during model runs. Every species has a maximum ID distance to the diver (in meters), within which they can be seen and correctly identified (and therefore counted). There is also a maximum approach distance (m), which is the distance to divers or predators that triggers evasive movement. Another important attribute is detectability, which is the probability of a fish being visible to the diver. Fish visibility is determined in every behaviour change (10 model seconds by default). A detectability below 1 means fish can become hidden from the diver when a behaviour change occurs, even if within ID distance (e.g. to simulate cryptic behaviour or mimicry).

Fish sensing capabilities are described by a perception distance (m) and a perception angle (0 - 360 degrees), which encompasses short distance detection of visual, tactile and chemical stimuli. A Boolean attribute establishes if fish will exhibit schooling behaviour. If true, a distance to schoolmates (in body lengths) must be specified and a list of schoolmates (conspecifics within perception angle and distance) is updated every time step for every fish. Fish state variables include their x and y coordinates, heading (degrees), the x and y components of their velocity vector and the x and y components of their acceleration vector. The magnitude of the vectors is limited by three attributes: maximum sustained speed (maximum velocity magnitude for continuous movement), maximum burst speed (maximum velocity magnitude in evasive movement) and maximum acceleration (maximum increase in speed that can occur in a second).

In order to calculate the acceleration vector at each time step, the model uses an urge-based movement algorithm that turns different “urges” (avoid diver, align with schoolmates, centre position in school, schoolmate spacing, wander, rest, cruise) into acceleration vectors. The magnitude of the urge vectors can be weighted to rank them in terms of importance, and these weights are stored as fish attributes. Another important fish attribute for the movement model is a constant used to estimate friction drag that is calculated from fish size and creates a deceleration vector opposite to the velocity vector at each movement cycle (see section 7.3). A set of weights for every urge defines a behaviour, and fish can have up to four behaviours stored in a list, each with an associated probability.

Finally, because some behaviours may require a fixed location in the environment (e.g. feeding, nesting), fish can pick a patch ahead of them and this patch is also stored in a variable, as well as the maximum distance (in meters) they can move away from it.

3) Process overview and scheduling

Every model run represents a single sample, using a single method of choice and sampling for a given time or distance. A cycle starts with a check to see if the diver has reached the end conditions of the sample. If the diver is finished, then outputs are calculated and the model run ends. If not, the model cycle continues.

The model cycle starts with the diver counting the fish that are eligible (see section 7.2), starting from the closest fish up to the number set as the count saturation (3 by default), which happens every second in model time. Then all the agents move with a pre-defined time step that can be set to tenths or fifths of a model second (default is 10 movement cycles per model second). The diver is the first agent to move, followed by

the buddy (if present), then all the fish move in an order that is randomly picked each cycle, regardless of species. In its turn to move, a fish will perform all vector calculations, determine the new velocity vector and move to the new position, then the next fish is picked.

When the movement cycle ends, fish listed on the diver's memory that left the field of view are removed from the list. Every time the model seconds match a multiple of 10 (by default) all fish must pick a behaviour from the behaviour list. This is done in a random order, but the first fish in a school to pick a behaviour will pick it for the whole school. In case there are fish with detectability smaller than 1, a Bernoulli trial is made for each fish in a random order to determine if it will be visible to the diver for the next 10 seconds (see behaviour change sub-model in section 7).

The sequence of events and sub-models on every time step is represented in a diagram in figure 1.

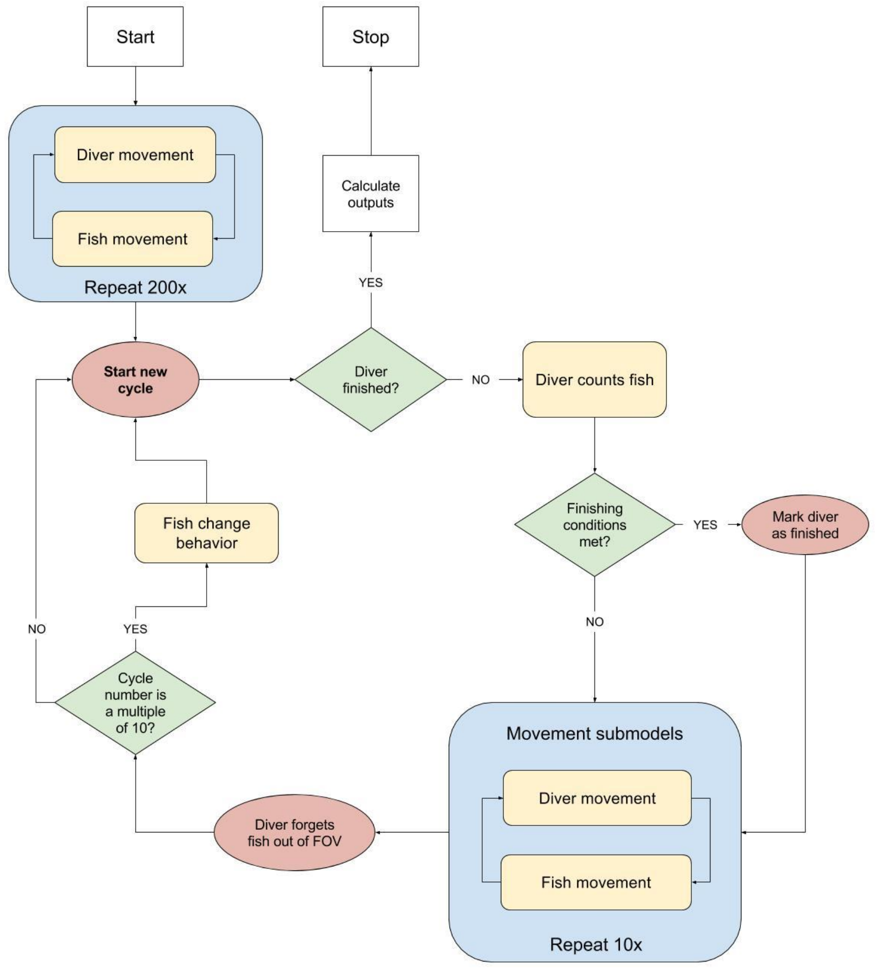

Figure 1. Overview of a cycle in the FishCensus model, representing 1 second in real time. Legend: FOV- Field of view.

4) Design concepts

4.1) Basic principles

The model applies what has been defined by Zurell et al. (2010) as a virtual ecologist approach, where not only the system is modelled, but also the sampling method. The model builds upon two previous visual census models, the Reefex model by Watson et al. (1995) and the AnimDens model by Ward-Paige et al. (2010). The simulation of sampling methods (timed transect, stationary point count and random path) is based on the AnimDens model, with the addition of a fixed distance transect, while the inclusion of diver avoidance, behaviour changes and count saturation is based on the Reefex model. When compared to both these models, FishCensus features a greater spatial and temporal resolution and more complex and realistic fish movement. The movement model is based on the implementation by Spector et al. (2005) of Craig Reynolds' boids (Reynolds, 1987), which uses a set of urges to calculate velocity vectors in groups of animals with collective movement. The specific implementation in NetLogo builds upon code by (Wilensky, 2005), with adaptations. Some important changes made to the movement algorithm are the inclusion of drag force that depends on the size and speed of the fish and the addition of new urges. In fact, while flocks of birds or swarms of insects tend to be in constant movement, fish are very often stationary, even when in schools. The FishCensus model intends to provide more realism in behaviour patterns and stochasticity, while including common sources of observation error.

4.2) Emergence

Fish movement, particularly schooling behaviour, emerge from the direction and magnitude of the urge vectors and the perception distance and angle of each species. Schools are in constant mutation and its members can vary. A school can be divided or even merged with other school, while each member is only aware of his closest neighbours and is unaware of school size. The size and shape of schools and their collective movement is emergent and is a consequence of perception angle and distance, schooling distance, maximum speed and acceleration, as well as the weight given to schooling-related urges (align, centre and spacing).

Ultimately, the model output (counts made by the divers in the simulated method) is a consequence of diver and fish attributes, position and behaviours, their interaction, water visibility and species detectability.

4.3) Adaptation

While behaviour parameters are constant throughout the model run, the maximum burst speed is only available to fish in the presence of a threat (diver) within the approach distance. When the threat is detected, the maximum speed a fish can reach increases until the threat is no longer in the perception range.

The fish movement algorithm is based on urges, therefore is strongly linked to the entities surrounding each fish. At every tenth of a model second, each fish is weighting all urges to decide on the next move. Although the outcome comes from a simple sum of vectors and is not subject to choice, a sudden change in scenario can lead to a drastic change in behavioural response.

4.4) Sensing

Sensing in the model ignores the size of agents and treats them as points with x and y coordinates. Agent A senses agent B only if the coordinates of agent B fall within the sensing area of agent A, which is also measured from the coordinates of A.

Both the diver and fish can sense their surroundings. The area the diver can perceive is defined by the view angle and maximum visibility. For fish within this area, the diver has access to their unique ID number (for memory storage) and species (for counts). Fish that can be counted are further constrained by their ID distance, whether they are visible at a time step (based on detectability) and sampling unit limits (transect borders or stationary point count radius).

Fish can sense an area delimited by perception distance, perception angle and approach distance, which can be set differently for every species. Fish perception is not affected by water visibility. Within perception distance, fish have access to the species, unique ID, heading, and velocity attributes of other fish, so they can identify schoolmates and adjust their state variables. Approach distance is used as a range at which fish detect the diver.

4.5) Interaction

All interactions in the model are direct and based on sensory attributes. If schooling is enabled for a species, every fish in a school can interact with conspecifics within perception distance and angle, by centring its position while attempting to keep a distance from schoolmates, avoiding collisions and aligning the direction of movement. If a diver enters the area delimited by approach distance and perception angle, a fish

can exhibit evasive behaviour, be indifferent or attracted, based on the weight given to the “avoid diver” urge (see section 7.3).

4.6) Stochasticity

Since a very important part of the model is to simulate variability in survey estimates, stochasticity is present in several processes. However, there is an option to use a fixed seed for the pseudo-random number generators so that every model run is identical. This can be useful to test different methods against a similar fish movement model run, however, changes in fish movement due to diver influence can be different in this case.

Fish are deployed with random x and y coordinates and the order in which they perform actions is sequential but randomised in every movement cycle. Furthermore, there is a random component in the fish movement sub-model (wander urge) that simply adds an acceleration vector with x and y components drawn from a uniform distribution between -1 and 1.

Fish behaviour changes are simplified in the model and the external or internal causes for change are ignored and simply translated into a deterministic behaviour change interval (in time steps) at which a weighted random selection with replacement occurs to pick one of up to four behaviours with pre-defined probabilities and parameters.

Diver movement is usually deterministic, except in the random path method, where the diver turns at a random angle (left or right) every two model seconds, up to the maximum value defined in the sampling parameters.

4.7) Collectives

The diver is a single agent, representing the only human agent and acting as the virtual ecologist, the observer.

Fish collectives are hierarchical. All “fish” agents share the exact same attributes and state variables, however, they can belong to different “species”, which can have different values for each attribute. The species collective can be used to separate fish agents into any homogeneous group, such as a real or theoretical species, a group of species sharing a certain behaviour pattern or different size groups of the same species. Within a species, there are two types of emergent collectives, schools and schoolmates.

If a fish exhibits schooling behaviour, all conspecifics within its perception area at any step in the movement cycle are grouped as “schoolmates”. This temporary and emergent collective can then be used by the fish to define its new heading, position

and speed (see section 7.3). Because each schoolmate of a given fish can have its own schoolmates, this can generate a school, a larger group which is never defined explicitly, but can be recognized visually by emergent coordinated movement.

4.8) Observation

The model fits into what has been generically called a virtual ecologist approach (Zurell et al., 2010), where the diver (i.e. the “virtual ecologist”) observes and records virtual fish, while performing a simulated sampling method. In the end of the sampling method, the counts made by the diver are divided by the sample area to calculate estimated densities. In order to calculate the accuracy of the sampling procedure, as well as the bias due to non-instantaneous observation, the real density of every species in the model environment is registered, as well as the density of each species inside the sampling area at the start of the sampling method.

For every species, the model outputs the real density, the estimated density (using the virtual ecologist) and bias due to non-instantaneous sampling. Bias is calculated as the difference between the estimated (non-instantaneous) density and the initial density inside the sample area, divided by the initial density (Ward-Paige et al., 2010). Therefore, a bias of 1 means that the diver counted on average one more fish per square meter for each fish it would have counted if sampling was instantaneous.

5) Initialization

Model initialization requires an input dataset with all attribute values for at least one species. All global variables are initialized or calculated and the parameters of the sampling method of choice are loaded. Sample areas for the method of choice are calculated (width x distance for transects, π * radius^2 for point counts).

Fish are placed on the environment with random coordinates and headings and both velocity and acceleration vector components set to 0. The number of fishes to place per species is calculated from a pre-defined true density. All attributes are set per species and all fish are visible, then all fish pick the first behaviour in the behaviour list, set behaviour parameters and set visibility if detectability is not 1. This first behaviour should be the most frequent or typical for the species. The movement model is then run for 200 cycles (20 model seconds) to stabilize the starting positions for fish (form schools, establish territories, etc.). Further behaviour changes do not occur during this stabilization phase and the model clock is not advanced.

The diver is then placed on the environment. For the stationary point count method, the diver is placed in the centre of the world, for transects, the diver starts on the centre of the 6th patch from the bottom, at the same distance from both margins of the world. Diver starts with a heading of 0 degrees for both methods. After the diver is placed, it sets memory and counted fishes as empty lists and a count of all fishes present in the sample area is stored to calculate theoretical instantaneous density estimates at time 0 (number of fish of a species / sample area).

6) Input data

The model does not use input data to represent time-varying processes.

7) Sub-models

7.1) Diver movement

Diver movement is much simpler than fish movement, however, since the position of the diver can influence the response of fish, it also moves every tenth of a model second (by default), even though counts are only made every second (see fish counting sub-model).

The movement sub-model differs with the chosen sampling method. For fixed distance transects, the diver maintains a heading of 0 degrees and moves forward at a pre-defined constant speed. For stationary point count, the diver starts with heading 0 degrees and rotates clockwise at a pre-defined constant speed (in degrees/second). For random path sampling, the diver swims forward at a fixed speed and turns at a random angle (left or right) every two model seconds, up to a maximum value.

The default diver movement models and parameters for the stationary point count and random path methods were adapted from Ward-Paige et al. (2010), with added spatial and temporal resolution. Transect diver swim speeds were derived from average swim speeds in real transects on temperate reefs (Pais et al., 2014). Nevertheless, speeds and sample area size can be changed freely.

7.2) Fish counting

This is a diver procedure that simulates the observation and recording of fish species during the virtual sampling method. Some parts of the counting sub-model differ with the chosen sampling method.

At every second in model time, the diver lists eligible fish that meet the following criteria:

1) Their coordinates fall within the field of view (defined by view angle and maximum visibility);
2) Their coordinates fall within the sample area (transect length and width or point count radius);
3) They are closer than their ID distance (distance at which an individual is identifiable);
4) They are visible (Boolean variable based on detectability);
5) Their unique ID is not in the diver's memory (only new or forgotten fish can be counted).

If the number of new fish is greater than the count saturation (three by default), then priority is given to the three fish standing closest to the diver. The "species" value of the new fishes is then added to the diver's list of counted fish and their unique ID is added to the memory list. Counted fish remain recorded until the end of the sample, but fish in the memory list are removed from memory if they leave the diver's field of view (allowing for recounts).

7.3) Fish movement

The parameterisation of the movement model at such a small scale relies heavily on real time observation of model runs and comparison with field observations or video footage, which is made easy by a user interface with sliders to observe changes. Parts of the movement sub-model code are based on the NetLogo implementation by Wilensky (2005) of the flocking model for BREVE by Spector et al. (2005). A set of urges are translated into two-dimensional acceleration vectors of magnitude 1 m/s^2 that are multiplied by weight coefficients given to different urges. Finally, a vector representing deceleration due to drag (A_d) is added. All vectors are then summed to generate a resultant acceleration vector (A) that is added to the velocity vector from the previous cycle to generate the new velocity vector:

$$A = \sum_{i=1}^{8} (w_i \, .A_i) + A_d$$

, where w_i and A_i are the weight and the acceleration vector for urge i. The magnitude of the velocity vector is limited by two attributes, the maximum cruise speed and the maximum burst speed. These are very important values for species movement simulation and can either be based on real measurements or estimated from the

caudal fin aspect ratio and total length (Sambilay Jr, 1990). The acceleration vector is also limited to a maximum value Blindly fitting a value here is difficult and may lead to unrealistic movement.

If the magnitude of the resultant acceleration vector exceeds the maximum acceleration, it is scaled down to this value and then added to the velocity vector on the previous cycle to calculate the x and y components of the new velocity. To avoid erratic movement at low speed, a minimum speed threshold was arbitrarily set at 0.2 m/s for all species in this study, below which no movement occurs.

The only time a velocity vector magnitude can exceed the maximum cruise speed is when a diver is within the approach distance and covered by the perception angle, and the diver avoidance urge weight is greater than zero. In this case, the maximum burst speed becomes the limit.

Fish can be stationary for long periods and often move with short bursts, followed by a coasting phase. This is a very important aspect of fish movement and is greatly influenced by drag forces (Videler, 1981). Since length is constant for a species in the model, the magnitude of the acceleration due to drag in a movement cycle can be written in the form $a_d = k\ v^2$, with v being the speed on the previous cycle. The constant k is calculated from the total length (L) in meters using the formula

$$k = \frac{\frac{1}{2} \times D \times d \times cL^2}{aL^b}$$

, where D is the drag coefficient, d is the density of the fluid (1027 kg/m^3 for surface seawater), c is the coefficient for the length - surface area relationship and a and b are coefficients for the length - weight relationship (see Appendix at the end of this document for details on the calculation). The coefficients a, b and c must be converted so the formulas reflect relationships in meters and kilograms. This formula establishes the magnitude of the acceleration vector, while its direction is always opposite to the velocity vector on the previous cycle.

While the values to estimate drag forces can be taken from real values measured for each species in the model, they are only available for a reduced number of species, and when using generic fish groups, it can be difficult to opt for one value over another. Given this, the extensively studied movement of the cod *Gadus morhua* Linnaeus, 1758 was used as an approximation for simplicity. Videler (1981) estimated that the drag coefficient of a coasting cod with 0.3 m total length is approximately 0.011. The length-weight relationship for cod can be converted to Kg and m from Coull et al.

(1989) to W= 10.3 $L^{2.857}$ and the coefficient for the length – surface area relationship (in metres) is approximately 40 (O'Shea et al., 2006).

A fish can have up to 8 urges acting simultaneously (table 1) and all vectors are normalized (given magnitude 1) before multiplying by weight coefficients (equation 1). Weights define the relative importance of urges and are characteristic of species, with a set of urge weights defining a behaviour for a species (see section 7.4). Weight for the diver avoidance urge can be set to negative values to simulate attraction to divers.

Table 1. Detailed description of the urge vectors used in the fish movement model.

**Urge**	**Description**	**Vector calculation**
Wander	Urge to move around randomly.	x and y components drawn from a uniform distribution between -1 and 1.
Cruise	Urge to maintain current heading.	Vector in the direction of velocity on the previous cycle.
Rest	Urge to stop moving.	Vector in the opposite direction of velocity on the previous cycle.
Align	Urge to align with schoolmates.	Mean of the x components and y components of the velocity vector of schoolmates.
Spacing	Urge to move away from schoolmates that are too close.	Sum of the vectors pointing away from schoolmates that are closer than the schooling distance, their magnitude being equal to the distance to each schoolmate.
Center	Urge to center the position relative to schoolmates.	Vector in the direction of the point defined by averaging x and y coordinates of all schoolmates.
Avoid diver	Urge to move away from the diver. Urge weight can be negative for attraction to divers.	Vector in the opposite direction of a diver who enters the area defined by approach distance and perception angle.
Patch center	Urge to move to the center of the picked patch if a fish moves outside the picked patch distance.	Vector in the direction of the picked patch center.

7.4) Fish behaviour change

The way behaviour change is implemented in FishCensus is based on the the Reefex model by Watson et al. (1995), including the default 10 second time step. In this case, however, a greater realism is achieved with a much smaller time step (tenths of a second) for movement, while behaviours still change every 10 seconds. This seems like a reasonable value for a parameter that must be arbitrarily set, given that behaviours are independent of external stimuli.

Attributes for up to four behavioural states, their names and frequencies are stored as fish variables. Stored attributes for a behavioural state are detectability, schooling (Boolean), schooling distance, urge weights (align, centre, spacing, wander, rest, cruise, patch gathering, diver avoidance) and picked patch distance.

Behavioural state frequencies are used every 10 model seconds to perform a weighted random selection with replacement. Every fish, in a randomized order, picks the next behaviour. If a fish has schoolmates, it will act as a leader and the others will immediately pick the same state, even if some of them may already have picked on that turn.

Once the next behaviour is picked, and if detectability is smaller than 1, the fish runs a Bernoulli trial to determine if it will be visible to the diver for the next 10 model seconds. This Bernoulli trial occurs with every behaviour change, always in a randomized order, while detectability remains smaller than 1. Visible status in schools is independently set for every fish.

The last step of the behaviour change sub-model takes place only if the weight given to the patch gathering urge is greater than 0. If this is the case, then it is assumed that the behavioural state requires a fixed patch. If a fish has not picked a patch on a previous state or commanded by a schoolmate on the present turn, it must choose the patch that stands 2 meters ahead. Once more, if there are schoolmates, they will skip the queue and pick the same patch immediately. If the weight given to the patch gathering urge is 0, the picked patch fish variable is cleared or replaced with a null value.

The behaviour change turn ends when all fish have picked the next behavioural state, set their visible status (if applicable) and picked a patch (if applicable).

8) Sensitivity analysis

To understand how sensitive the output of the model is to small changes in parameters, a local sensitivity analysis was run using 30x2 m transects, a diver swimming at 8 m per minute with a visibility of 6 metres and true density of fish fixed at 0.2 fish per m^2. 15 replicates were run for each parameter.

For this analysis, five fish types were created (tables 2 and 3): A basic type without schooling behaviour or urge to rest and detectability set to 1, a variation of this type with schooling behaviour to test changes in schooling parameters, a second variation with 50% detectability to test changes in this parameter and a third variation with a rest urge of 5 to test changes in the magnitude of this vector. The fifth type is a complex schooling species with three behaviours, used to test behaviour change interval and frequencies (table 3).

Behaviour change interval (10 seconds) and count saturation (3 fish per second) are two structural parameters of the model (even though they can be changed in the interface), which had to be somewhat arbitrarily set.

The “Complex” fish type was used to test the sensitivity to behaviour change interval (tables 2 and 3). Increasing behaviour change interval by 1 second (10%) led to an average increase of 15.2% (8.3% SE) on the estimated density, while decreasing this parameter by 1 second led to an increase of 4.1% (7.2% SE). These values suggest the output is relatively robust to small changes in this model simplification parameter. For the count saturation, counting 1 less fish per minute led to an increase in 0.4% in the output (3.8% SE) and counting one more fish led to a 1.6% increase (4.0% SE).

Table 2. Fixed attributes for the five generic fish types used as controls for sensitivity analysis. The four basic fish types have similar fixed attributes and differ only on the behaviour parameters (table 3).

	Basic (4 types)	Complex (1 type)
Size (m)	0.3	0.2
ID distance (m)	5	4
Approach distance (m)	1.0	1.0
Perception distance (m)	–	0.35
Perception angle (degrees)	320	320
Max. acceleration (m/s^2)	0.15	0.2
Max. sustained speed (m/s)	0.5	0.5
Burst speed (m/s)	2.9	2.6

The sensitivity of fish attributes and behaviour submodel parameters will likely be influenced by the combination of behaviours, frequencies, sampling method, visibility, among other factors, in a way that would be overwhelming and too computationally intensive to approach exhaustively. Instead, users of FishCensus are encouraged to look at sensitivity for their species and method of interest. The generic species used in this simple sensitivity analysis have a single behaviour with most urge vectors set to an intermediate value (5), so changing this value by an integer corresponds to a 20% variation of the input (figure 2).

The sensitivity of behaviour frequency was assessed by shifting the frequency of the "wander" behaviour of the complex fish by 0.1 in both directions, and diluting this change on the other two behaviours (table 3).

Table 3. Behaviour parameters for the five generic fish types used as controls for sensitivity analysis. The first four types are variations of the basic type, all with a single behaviour and with changes highlighted in bold. The fifth is a complex schooling species with three behaviours.

	Fish type name	Basic	School	Detectability	Rest	Complex		
	Behaviour name	basic	basic	basic	basic	wandering	feeding	stationary
	Frequency	1	1	1	1	0.5	0.2	0.3
	Detectability	1	1	**0.5**	1	1	1	1
	Schooling?	FALSE	**TRUE**	FALSE	FALSE	TRUE	TRUE	TRUE
	Schoolmate distance (BL)	–	1	–	–	1	1	1
	Patch distance (m)	–	–	–	–	–	1	–
Urge weights	Align	–	**5**	–	–	5	1	5
	Centre	–	**5**	–	–	6	2	6
	Spacing	–	**5**	–	–	15	5	15
	Wander	5	5	5	5	3	1	1
	Rest	0	0	0	**5**	0	1	7
	Cruise	5	5	5	5	0	0	0
	Patch gathering	–	–	–	–	–	10	–
	Diver avoidance	5	5	5	5	10	10	10

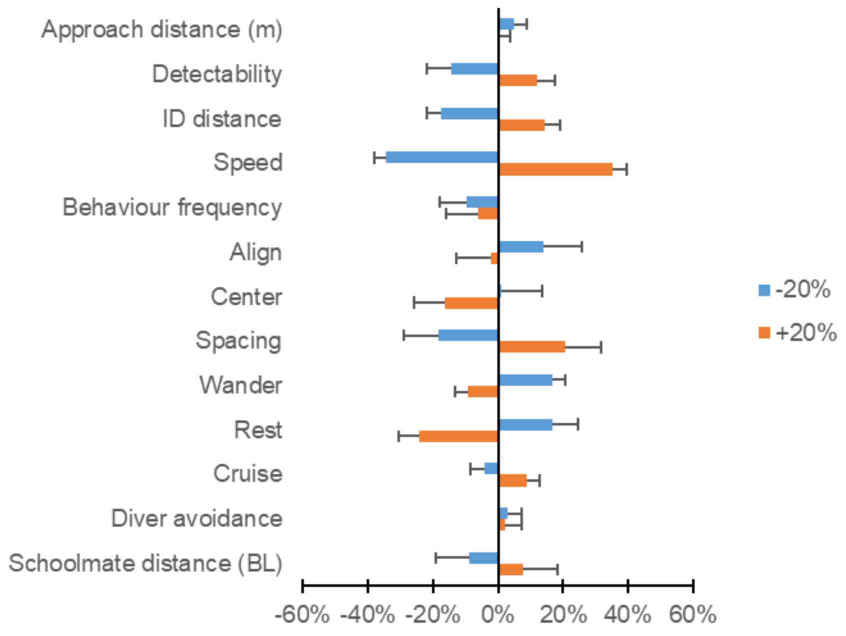

Figure 2. Percent change in output (fish density estimated by the diver) with an increase or decrease in 20% of the input parameters relative to control. Values calculated from 15 replicates using 30x2 m transects, with a swim speed of 8 metres per minute and a visibility of 6 metres. True density was fixed at 0.2 fish per square metre.

For the speed attribute, the cruise speed was shifted 20% in both directions and the burst speed was also adjusted by the same amount (not the same percentage). Even though this is a simple approach that disregards all possible interactions among parameters, it is evident from figure 2 that the model is particularly sensitive to fish movement speed (maximum speed and the magnitude of the rest urge). The rest urge adds additional drag to fish movement, regardless of their maximum speed, so changing this urge can drastically change the average speed and thus impact the visual counts. Maximum speed, however, is one of the most important attributes in the model, requiring a particularly informed parameterisation, either from laboratory measurements, video analysis or by using a proxy such as the caudal fin aspect ratio approach implemented in the model interface.

For schooling behaviour, the output seems to be particularly sensitive to the spacing between schoolmates. Decreasing or increasing the distance by 20% led to a decrease or increase in output by about 20%, respectively. Because increasing the “centre” urge vector leads to more compact schools, it is analogous to decreasing the distance in terms of sensitivity.

The effect of changing fish view angle was also tested, but as in this case it is impossible to increase it by 20%, this parameter was tested by reducing or increasing the angle in 10 degrees (3%). In this case a decrease led to a 2.0% average increase in estimated density (4.0% SE), while an increase led to an average increase of the output in 1.8% (3.9% SE). Because this was done on the "basic" fish type (tables 1 and 2), view angle only affects the reaction of fish to the diver. Changing the view angle on a schooling fish tends to affect the shape and size of schools, and therefore model sensitivity is probably affected differently.

References

Coull, K.A., Jermyn, A.S., Newton, A.W., Henderson, G.I., Hall, W.B., 1989. Length/Weight relationships for 88 species of fish encountered in the North East Atlantic, Scottish Fisheries Research Report. Aberdeen, Scotland.

Grimm, V., Berger, U., Bastiansen, F., Eliassen, S., Ginot, V., Giske, J., Goss-Custard, J., Grand, T., Heinz, S.K., Huse, G., Huth, A., Jepsen, J.U., Jørgensen, C., Mooij, W.M., Müller, B., Pe'er, G., Piou, C., Railsback, S.F., Robbins, A.M., Robbins, M.M., Rossmanith, E., Rüger, N., Strand, E., Souissi, S., Stillman, R.A., Vabø, R., Visser, U., DeAngelis, D.L., 2006. A standard protocol for describing individual-based and agent-based models. Ecol. Modell. 198, 115–126. doi:10.1016/j.ecolmodel.2006.04.023

Grimm, V., Berger, U., DeAngelis, D.L., Polhill, J.G., Giske, J., Railsback, S.F., 2010. The ODD protocol: A review and first update. Ecol. Modell. 221, 2760–2768. doi:10.1016/j.ecolmodel.2010.08.019

Luck, S.J., Vogel, E.K., 1997. The capacity of visual working memory for features and conjunctions. Nature 390, 279–281.

O'Shea, B., Mordue-Luntz, A.J., Fryer, R.J., Pert, C.C., Bricknell, I.R., 2006. Determination of the surface area of a fish. J. Fish Dis. 29, 437–440. doi:10.1111/j.1365-2761.2006.00728.x

Pais, M.P., Henriques, S., Costa, M.J., Cabral, H.N., 2014. Topographic complexity and the power to detect structural and functional changes in temperate reef fish assemblages: The need for habitat-independent sample sizes. Ecol. Indic. 45, 18–27. doi:10.1016/j.ecolind.2014.03.018

Reynolds, C.W., 1987. Flocks, herds, and schools: A Distributed behavioral model. Comput. Graph. (ACM). 21, 25–34.

Sambilay Jr, V.C., 1990. Interrelationships between swimming speed, caudal fin aspect ratio and body length of fishes. Fishbyte 8, 16–20.

Spector, L., Klein, J., Perry, C., Feinstein, M., 2005. Emergence of collective behavior in evolving populations of flying agents. Genet. Program. Evolvable Mach. 6, 111–125. doi:10.1007/s10710-005-7620-3

Videler, J.J., 1981. Swimming Movements, Body Structure and Propulsion in Cod Gadus morhua. Symp. zool. Soc. Lond. 48, 1–27.

Ward-Paige, C.A., Flemming, J.M., Lotze, H.K., 2010. Overestimating fish counts by non-instantaneous visual censuses: Consequences for population and community descriptions. PLoS One 5, e11722. doi:10.1371/journal.pone.0011722

Watson, R.A., Carlos, G.M., Samoilys, M.A., 1995. Bias introduced by the non-random movement of fish in visual transect surveys. Ecol. Modell. 77, 205–214. doi:10.1016/0304-3800(93)E0085-H

Wilensky, U., 2005. NetLogo flocking 3D alternate model. http://ccl.northwestern.edu/netlogo/models/Flocking3DAlternate. Center for Connected Learning and Computer-Based Modeling, Northwestern University, Evanston, IL, USA.

Zurell, D., Berger, U., Cabral, J.S., Jeltsch, F., Meynard, C.N., Münkemüller, T., Nehrbass, N., Pagel, J., Reineking, B., Schröder, B., Grimm, V., 2010. The virtual ecologist approach: Simulating data and observers. Oikos 119, 622–635. doi:10.1111/j.1600-0706.2009.18284.x

Appendix

Calculation of acceleration due to drag on the FishCensus fish movement sub-model

This is a summary of the calculations. For references and details on how these calculations fit in the model, see the full ODD description above.

Since the observed acceleration of an actively swimming fish already considers drag forces, it is the coasting phase that is of interest for the movement model. The drag force (F_d) acting over a coasting fish is calculated as:

$$F_d = \frac{1}{2} \times D \times d \times A_s \times v^2 \quad (1)$$

, where D is the drag coefficient, d is the density of the fluid (1027 kg/m^3 for surface seawater), v is the speed and A_s is the wetted surface area.

In order to calculate acceleration from the drag force, the fish body mass must be taken into account. Since length is an attribute of fish in the model, the weight (W) in kilograms can be estimated from a length-weight relationship:

$$W = a\,L^b \quad (2)$$

, where L is the total length in metres.

For simplicity, the surface area of a fish can be roughly estimated as a function of total length squared, multiplied by a coefficient that varies with body shape:

$$A_s = c\,L^2 \quad (3)$$

Given that $F_d = W a_d$, and substituting W by the length-weight relationship, the magnitude of the acceleration due to drag (a_d) in a movement cycle can be written in the form:

$$a_d = k\,v^2 \quad (4)$$

, with v being the speed on the previous cycle. The constant k is calculated from the total length (L) in meters using equations 1, 2 and 3:

$$k = \left(\frac{1}{2} \times D \times d \times cL^2\right) / \left(aL^b\right) \quad (5)$$

, where D is the drag coefficient, d is the density of the fluid, c is the coefficient for the length - surface area relationship and a and b are coefficients for the length - weight relationship. The coefficients a, b and c must be converted so the formulas reflect relationships in meters and kilograms.
