# Supplementary material for: Effect of underwater visual survey methodology on bias and precision of fish counts: a simulation approach
Source: PeerJ. 2018 Jul 30;6:e5378. doi: 10.7717/peerj.5378 (PMC6071614; doi:10.7717/peerj.5378)
Supplement: Table S1 — Detailed summaries for the multiple linear regression models for all fish types. (A) Summary tables for point counts. (B) Summary tables for transects. [file peerj-06-5378-s007.pdf]

Table S1.A. Multiple linear regression models for point counts

Schooling: sqrt(bias) ~ radius * rot_speed * time

	Estimate	Std. Error	t value	Pr(>|t|)	
(Intercept)	1.8511476	0.3378799	5.479	5.43e-08	***
radius	-0.2716336	0.0919593	-2.954	0.00321	**
rot_speed	0.0353690	0.0509373	0.694	0.48762	
time	0.3349372	0.0447533	7.484	1.59e-13	***
radius:rot_speed	0.0093044	0.0138634	0.671	0.50228	
radius:time	-0.0272708	0.0121803	-2.239	0.02538	*
rot_speed:time	0.0095302	0.0067468	1.413	0.15810	
radius:rot_speed:time	-0.0009021	0.0018362	-0.491	0.62334	

Residual standard error: 0.5194 on 992 degrees of freedom
Multiple R-squared: 0.7853, Adjusted R-squared: 0.7838
F-statistic: 518.4 on 7 and 992 DF, p-value: < 2.2e-16

Cryptic: sqrt(bias) ~ log(radius) * rot_speed * time

	Estimate	Std. Error	t value	Pr(>|t|)	
(Intercept)	-0.3673595	0.2414430	-1.522	0.1284	
log(radius)	0.9308536	0.1939395	4.800	1.83e-06	***
rot_speed	0.0619799	0.0363989	1.703	0.0889	.
time	0.1561368	0.0319799	4.882	1.22e-06	***
log(radius):rot_speed	-0.0508950	0.0292375	-1.741	0.0820	.
log(radius):time	-0.1237475	0.0256879	-4.817	1.68e-06	***
rot_speed:time	0.0014740	0.0048212	0.306	0.7599	
log(radius):rot_speed:time	-0.0008057	0.0038726	-0.208	0.8352	

Residual standard error: 0.3356 on 992 degrees of freedom
Multiple R-squared: 0.2035, Adjusted R-squared: 0.1979
F-statistic: 36.21 on 7 and 992 DF, p-value: < 2.2e-16

Shy: bias ~ radius * rot_speed * time

	Estimate	Std. Error	t value	Pr(>|t|)	
(Intercept)	0.657394	1.460111	0.450	0.6527	
radius	-0.360010	0.357653	-1.007	0.3145	
rot_speed	-0.223403	0.220120	-1.015	0.3105	
time	1.480154	0.193396	7.653	6.09e-14	***
radius:rot_speed	0.058169	0.053918	1.079	0.2810	
radius:time	-0.085590	0.047372	-1.807	0.0712	.
rot_speed:time	-0.073919	0.029156	-2.535	0.0114	*
radius:rot_speed:time	0.034602	0.007142	4.845	1.54e-06	***

Residual standard error: 1.278 on 742 degrees of freedom
Multiple R-squared: 0.9306, Adjusted R-squared: 0.9299
F-statistic: 1421 on 7 and 742 DF, p-value: < 2.2e-16

Bold: log(bias) ~ radius * rot_speed * time

	Estimate	Std. Error	t value	Pr(>|t|)	
(Intercept)	3.406e+00	1.146e-01	29.718	<2e-16	***
radius	-4.937e-01	3.119e-02	-15.828	<2e-16	***
rot_speed	1.037e-02	1.728e-02	0.600	0.5486	
time	1.570e-01	1.518e-02	10.340	<2e-16	***
radius:rot_speed	1.091e-02	4.702e-03	2.320	0.0205	*
radius:time	4.124e-03	4.132e-03	0.998	0.3184	
rot_speed:time	-2.807e-05	2.288e-03	-0.012	0.9902	
radius:rot_speed:time	-1.102e-04	6.228e-04	-0.177	0.8596	

Residual standard error: 0.1762 on 992 degrees of freedom
Multiple R-squared: 0.936, Adjusted R-squared: 0.9355
F-statistic: 2072 on 7 and 992 DF, p-value: < 2.2e-16

Table S1.B Multiple linear regression models for transects

Schooling: sqrt(bias) ~ distance * width * speed

```
                      Estimate Std. Error t value Pr(>|t|)    
(Intercept)          2.736e+00  1.232e-01  22.205  < 2e-16 ***
distance             5.713e-03  3.714e-03   1.538    0.124    
width               -2.067e-01  3.714e-02  -5.564 3.23e-08 ***
speed               -1.730e-01  1.857e-02  -9.314  < 2e-16 ***
distance:width      -4.379e-04  1.120e-03  -0.391    0.696    
distance:speed      -4.962e-05  5.600e-04  -0.089    0.929    
width:speed          8.706e-03  5.600e-03   1.555    0.120    
distance:width:speed -3.553e-05  1.688e-04  -0.210    0.833    

Residual standard error: 0.3377 on 1242 degrees of freedom
Multiple R-squared:  0.6862,	Adjusted R-squared:  0.6845 
F-statistic: 388.1 on 7 and 1242 DF,  p-value: < 2.2e-16
```

Cryptic: bias ~ distance * width * speed

```
                      Estimate Std. Error t value Pr(>|t|)    
(Intercept)          3.586e-01  6.494e-02   5.522 4.08e-08 ***
distance            -8.771e-03  1.958e-03  -4.480 8.15e-06 ***
width                8.078e-03  1.958e-02   0.413  0.67999    
speed               -4.248e-03  9.789e-03  -0.434  0.66442    
distance:width       2.510e-03  5.903e-04   4.252 2.28e-05 ***
distance:speed       6.965e-04  2.952e-04   2.360  0.01843 *  
width:speed          8.573e-03  2.952e-03   2.904  0.00374 ** 
distance:width:speed -1.986e-04  8.899e-05  -2.231  0.02583 *  

Residual standard error: 0.178 on 1242 degrees of freedom
Multiple R-squared:  0.4472,	Adjusted R-squared:  0.444 
F-statistic: 143.5 on 7 and 1242 DF,  p-value: < 2.2e-16
```

Shy: sqrt(bias) ~ distance * width * speed

```
                      Estimate Std. Error t value Pr(>|t|)    
(Intercept)          3.5797120  0.1149468  31.142  < 2e-16 ***
distance             0.0191442  0.0034658   5.524 4.04e-08 ***
width               -0.3398881  0.0346578  -9.807  < 2e-16 ***
speed               -0.2095855  0.0173289 -12.095  < 2e-16 ***
distance:width      -0.0020290  0.0010450  -1.942   0.0524 .  
distance:speed      -0.0010842  0.0005225  -2.075   0.0382 *  
width:speed          0.0104006  0.0052249   1.991   0.0467 *  
distance:width:speed  0.0002315  0.0001575   1.470   0.1419    

Residual standard error: 0.3151 on 1242 degrees of freedom
Multiple R-squared:  0.8334,	Adjusted R-squared:  0.8325 
F-statistic: 887.6 on 7 and 1242 DF,  p-value: < 2.2e-16
```

Bold: log(bias) ~ log(distance) * log(width) * log(speed)

```
                                    Estimate Std. Error t value Pr(>|t|)    
(Intercept)                         4.016008   0.158439  25.347  < 2e-16 ***
log(distance)                       0.126352   0.047877   2.639 0.008418 ** 
log(width)                         -0.428782   0.142288  -3.013 0.002635 ** 
log(speed)                         -1.113838   0.090756 -12.273  < 2e-16 ***
log(distance):log(width)           -0.088245   0.042997  -2.052 0.040345 *  
log(distance):log(speed)            0.002144   0.027425   0.078 0.937713    
log(width):log(speed)              -0.303767   0.081504  -3.727 0.000202 ***
log(distance):log(width):log(speed)  0.067181   0.024629   2.728 0.006467 ** 

Residual standard error: 0.1599 on 1242 degrees of freedom
Multiple R-squared:  0.9651,	Adjusted R-squared:  0.9649 
F-statistic:  4900 on 7 and 1242 DF,  p-value: < 2.2e-16
```
