# Supplementary material for: Effect of underwater visual survey methodology on bias and precision of fish counts: a simulation approach
Source: PeerJ. 2018 Jul 30;6:e5378. doi: 10.7717/peerj.5378 (PMC6071614; doi:10.7717/peerj.5378)

Figure S1.A. Raw response of bias to point count method parameters. Each point in the line is averaged from 10 replicates. For these curves, bias is calculated as the difference (not absolute) between average estimated density and true density, divided by true density (0.3 fish/m^2). Radius of 1 m was not used in the analyses but its effect is shown here.

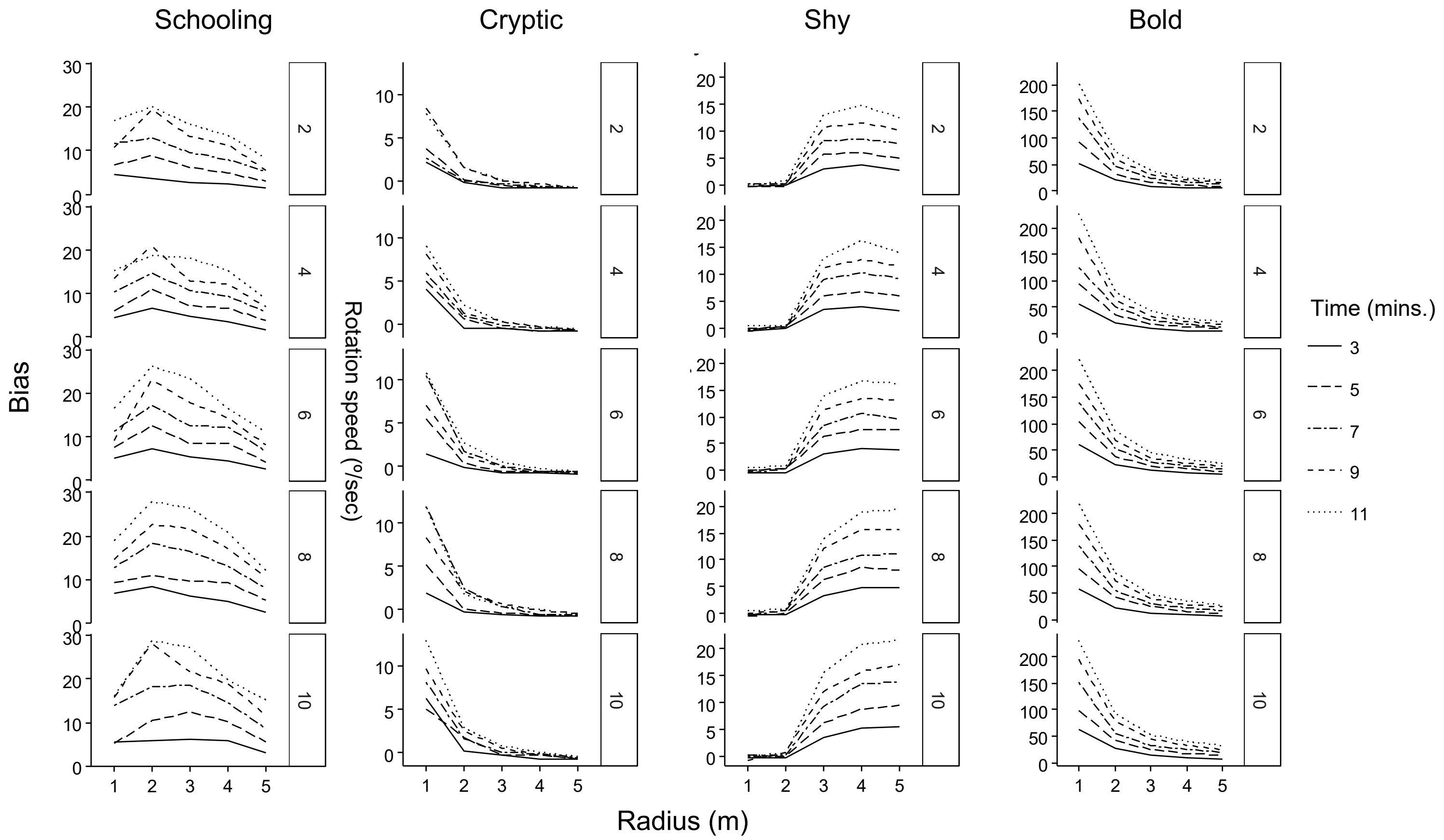

Figure S1.B. Raw response of the coefficient of variation (CV) to point count method parameters. Each point in the line is calculated from 10 replicates. CV is calculated as the standard deviation of estimates divided by true density (0.3 fish/m^2). Radius of 1 m was not used in the analyses but its effect is shown here.

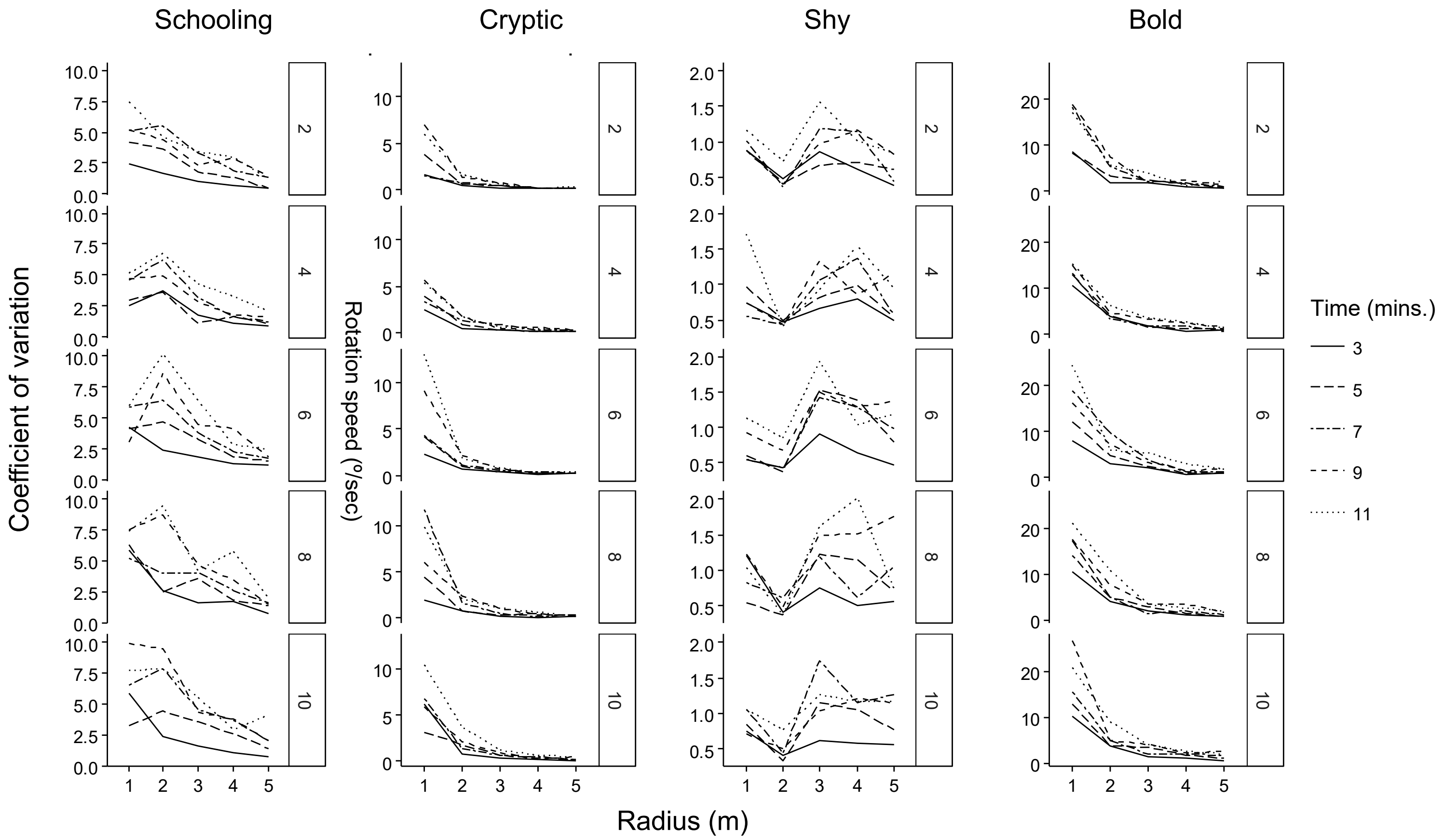

Figure S1.C. Raw response of bias to transect method parameters. Each point in the line is averaged from 10 replicates. For these curves, bias is calculated as the difference (not absolute) between average estimated density and true density, divided by true density (0.3 fish/m^2).

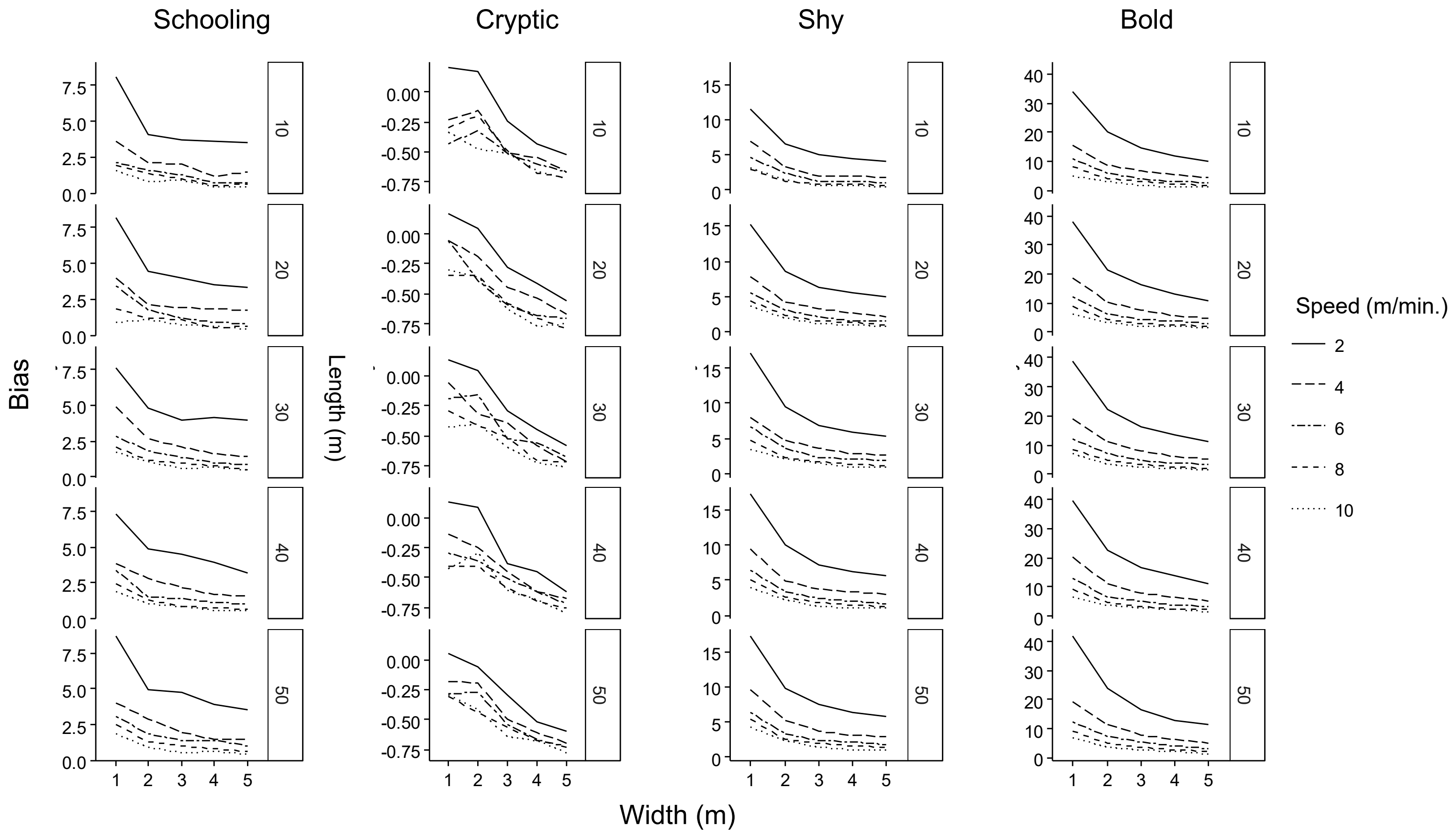

Figure S1.D. Raw response of the coefficient of variation (CV) to transect method parameters. Each point in the line is calculated from 10 replicates. CV is calculated as the standard deviation of estimates divided by true density (0.3 fish/m^2).

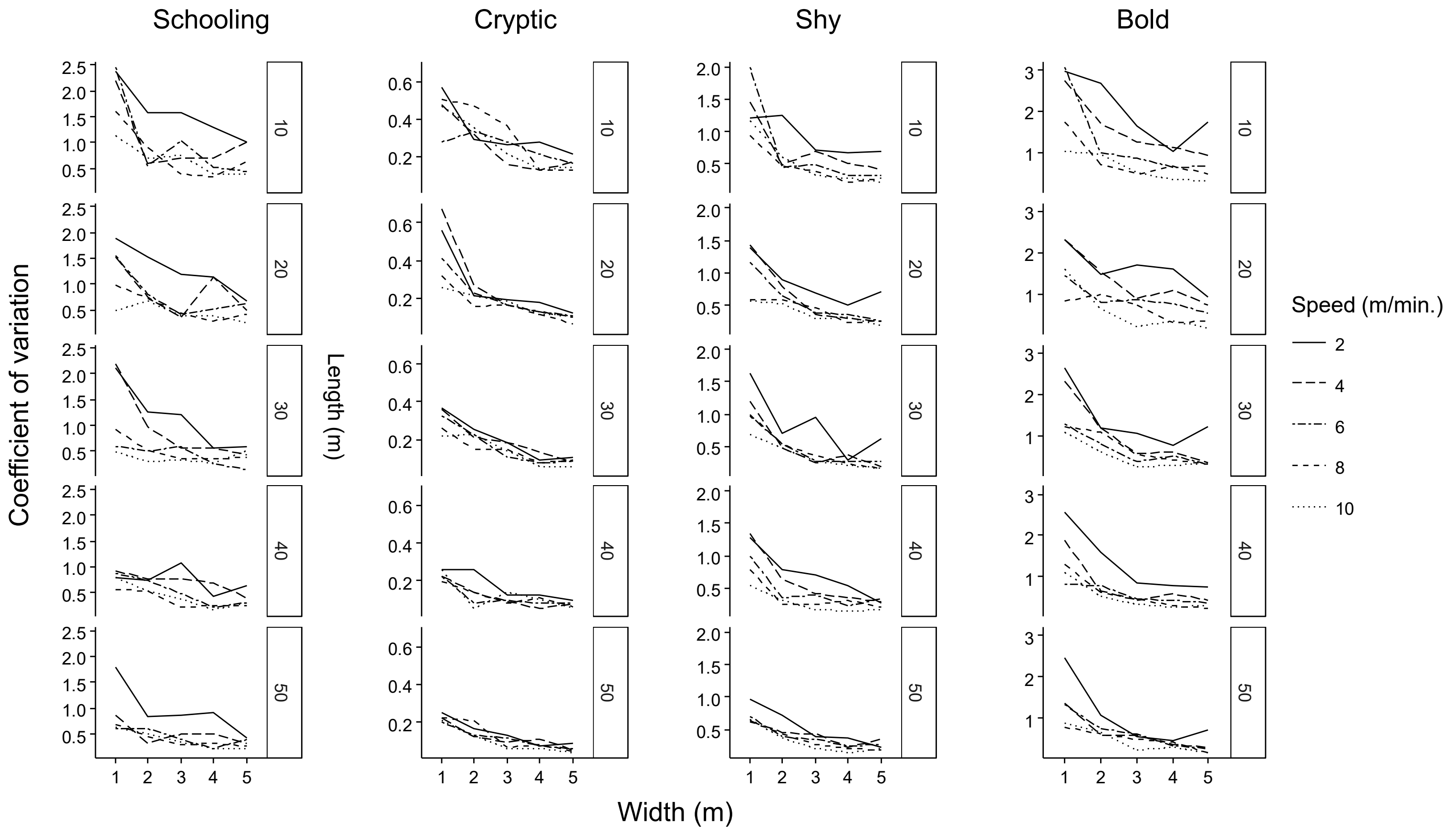

Supplement: Figure S1 — Average bias calculated from 10 replicates as the difference between estimated and true abundance, divided by true abundance. Positive bias corresponds to an overestimation of fish density. Precision calculated as the standard deviation of the 10 estimates, divided by true density. A radius of 1m for point counts was not used in the analyses but its effect is shown. (A) Bias in point counts. (B) Precision in point counts. (C) Bias in transects. (D) Precision in transects. [file peerj-06-5378-s008.pdf]
